# Resveratrol ameliorates intrahepatic cholestasis of pregnancy by modulating the gut-liver axis and FXR-mediated bile acid homeostasis

**DOI:** 10.3389/fimmu.2026.1819374

**Published:** 2026-05-18

**Authors:** Ningning Hu, Ying Yang, Rujun Chen, Junhua Guan, Huafen Gu, Lingling Zhang, Xuemei Zhang, Xiaoqin Wang, Liwen Zhang

**Affiliations:** Department of Obstetrics and Gynecology, Shanghai Fifth People’s Hospital, Fudan University, Shanghai, China

**Keywords:** bile acid homeostasis, farnesoid X receptor, gut-liver axis, intrahepatic cholestasis of pregnancy, resveratrol

## Abstract

**Objective:**

Intrahepatic cholestasis of pregnancy (ICP) is a liver disorder with limited treatment options. This study investigated the therapeutic potential of resveratrol (RES) and its underlying mechanisms, focusing on the gut-liver axis and bile acid metabolism in an estrogen-induced ICP rat model.

**Methods:**

Pregnant rats were randomized into Sham, ICP (induced by 17β-estradiol), and ICP+RES (15, 30, 60 mg/kg) groups. Systemic and hepatic inflammation, liver function, histopathology, and intestinal barrier integrity were assessed. Hepatic bile acid profiles were analyzed by UHPLC-MS/MS, and gut microbiota was evaluated by 16S rRNA sequencing. The role of gut microbiota was further examined via fecal microbiota transplantation (FMT) in pseudogerm-free rats. Key proteins in the FXR signaling pathway were analyzed by Western blotting.

**Results:**

RES treatment dose-dependently alleviated ICP manifestations, including reducing serum levels of total bile acids, total bilirubin, and liver enzymes (AST, ALT, ALP), while mitigating systemic and hepatic inflammation. It also restored intestinal barrier integrity and corrected gut microbiota dysbiosis. FMT from RES-treated donors recapitulated these therapeutic effects in recipient ICP rats. Furthermore, RES reversed the hepatic bile acid imbalance by reducing primary bile acids and increasing beneficial secondary bile acids. Mechanistically, RES upregulated the expression of FXR and its downstream targets, including SHP, BSEP, UGT2B4, and CYP1A1.

**Conclusion:**

RES effectively ameliorated ICP through multi-faceted mechanisms involving the attenuation of inflammation, restoration of gut microbiota and intestinal barrier, and correction of bile acid homeostasis via activation of the FXR signaling pathway. Our findings highlight RES as a promising multi-mechanistic therapeutic candidate for ICP.

## 1 Introduction

Intrahepatic cholestasis of pregnancy (ICP) is a clinically significant obstetric disorder characterized by disrupted bile acid homeostasis, leading to maternal pruritus and elevated risks of adverse fetal outcomes (1). The etiopathogenesis of ICP involves an intricate interplay between hormonal influences and dysregulated hepatobiliary transport, predominantly mediated through estrogen-induced suppression of key bile acid transporters (2, 3). Although ursodeoxycholic acid remains the current therapeutic mainstay, its constrained efficacy underscores the need for novel interventions targeting the fundamental mechanisms of cholestatic injury.

Accumulating evidence has elucidated the pivotal involvement of the gut–liver axis in cholestatic pathophysiology (4). The intestinal microbiota actively modulates bile acid metabolism through enzymatic biotransformation and directly impacts hepatic inflammation via bacterial translocation mechanisms (5, 6). Contemporary research demonstrates that gut microbial dysbiosis significantly influences farnesoid X receptor (FXR) signaling dynamics and bile acid pool composition, thereby propagating cholestatic progression (7, 8). Furthermore, compromised intestinal barrier integrity and consequent endotoxin translocation exacerbate hepatic inflammation through activation of pro-inflammatory cascades.

Resveratrol (RES), a naturally occurring polyphenol with documented hepatoprotective properties, has demonstrated therapeutic potential across various models of hepatic pathology (9). Its established mechanisms encompass anti-inflammatory activity, nuclear receptor signaling modulation, and intestinal barrier preservation (10). Nevertheless, its specific application in ICP management remains largely unexplored, particularly regarding its effects on gut–liver axis communication and bile acid metabolic regulation.

Currently, it remains unclear whether RES could exert multi-faceted anti-ICP effects by modulating gut microbiota structure, improving intestinal barrier function, regulating the FXR signaling pathway, and restoring bile acid metabolic homeostasis. To address this, the present study employed an estrogen-induced ICP rat model to comprehensively evaluate the impact of RES on systemic inflammation, liver function, intestinal barrier integrity, gut microbiota composition, and hepatic bile acid profiles. Furthermore, fecal microbiota transplantation was conducted to verify the causal role of gut microbes in the therapeutic efficacy of RES. At the molecular level, we investigated whether RES activates the FXR signaling pathway to regulate key transporters and metabolic enzymes, thereby ultimately restoring bile acid homeostasis. This study aims to systematically elucidate the multi-mechanistic protective effects of RES against ICP, providing a theoretical and experimental foundation for its clinical translation.

## 2 Materials and methods

### 2.1 Animals

Specific pathogen-free pregnant Sprague-Dawley rats (180–220 g) were procured from Beijing Vital River Laboratory Animal Technology Co., Ltd. (Beijing, China). Animals were housed under controlled conditions (12-h light/dark cycle, 22 ± 2 °C, 50 ± 5% humidity) with ad libitum access to standard chow and sterile water. All experimental protocols were approved by the Institutional Animal Care and Use Committee of Animal Experiment Center of Minhang Campus of East China Normal University (R20221204) and conducted in accordance with the NIH Guide for the Care and Use of Laboratory Animals.

### 2.2 Reagents and antibodies

17β-estradiol (EE2, ≥98%), RES (≥98%), dimethyl sulfoxide, ampicillin, neomycin, metronidazole, and vancomycin were sourced from Sigma-Aldrich (St. Louis, MO, USA). ELISA kits for lipopolysaccharide (LPS), interleukin-1β (IL-1β), total bilirubin (TBil), and monocyte chemoattractant protein-1 (MCP-1) were obtained from R&D Systems (Minneapolis, MN, USA). Primary antibodies against FXR, small heterodimer partner (SHP), bile salt export pump (BSEP), UDP glucuronosyltransferase family 2 member B4 (UGT2B4), cytochrome P450 family 1 subfamily A member 1 (CYP1A1), Na^+^–taurocholate cotransporting polypeptide (NTCP), and glyceraldehyde-3-phosphate dehydrogenase (GAPDH) were purchased from Cell Signaling Technology (Danvers, MA, USA), Abcam (Cambridge, UK), and Santa Cruz Biotechnology (Dallas, TX, USA). Horseradish peroxidase–conjugated secondary antibodies were acquired from Cell Signaling Technology. The QIAamp Fast DNA Stool Mini Kit (Qiagen, Hilden, Germany) and Agencourt AMPure XP Kit (Beckman Coulter, Brea, CA, USA) were used for DNA extraction and purification.

### 2.3 Establishment of the estrogen-induced ICP rat model and RES treatment protocol

Pregnant rats were randomly assigned to five experimental groups (n = 8 per group) on gestational day (GD) 17: Sham group: Rats received subcutaneous injections of normal saline (0.1 mL/100 g body weight) once daily from GD 17 to GD 21. Concurrently, intragastric gavage of vehicle (DMSO–normal saline mixture, final DMSO concentration ≤ 1%) was administered at the same volume as the RES treatment groups. ICP group: Rats were subcutaneously injected with EE2 (0.6 mg/kg/day) dissolved in DMSO once daily from GD 17 to GD 21 to induce ICP. Intragastric gavage of vehicle was given simultaneously. ICP + RES (15 mg/kg) group: EE2-induced ICP rats received intragastric administration of RES (15 mg/kg/day) once daily, starting on GD 17 and continuing until GD 21. RES was dissolved in DMSO and diluted with normal saline to the required concentration. ICP + RES (30 mg/kg) group: EE2-induced ICP rats were treated with RES at a dose of 30 mg/kg/day via intragastric gavage, following the same administration schedule as the 15 mg/kg group. ICP + RES (60 mg/kg) group: EE2-induced ICP rats were administered RES at 60 mg/kg/day via intragastric gavage, with the same administration timeline as the other RES treatment groups.

All rats were euthanized on GD 21 via cervical dislocation under isoflurane anesthesia. Serum samples were collected by centrifuging blood at 3000 × g for 15 min at 4 °C. Liver and intestinal tissues were excised immediately, rinsed with ice-cold phosphate-buffered saline (PBS), and either fixed in 4% paraformaldehyde for histopathological analysis or stored at –80 °C for subsequent biochemical and molecular experiments. Fresh fecal samples were collected under sterile conditions and stored at –80 °C for gut microbiota analysis.

### 2.4 Fecal microbiota transplantation in pseudogerm-free rats

Pregnant rats were randomly divided into three groups (n = 8 per group) on GD 10 to establish pseudogerm-free (PGF) models using antibiotic (ABX) treatment: ABX-PBS group: Rats were provided with drinking water containing a mixture of antibiotics (ampicillin 1 g/L, neomycin 1 g/L, metronidazole 1 g/L, vancomycin 0.5 g/L) from GD 10 to GD 21. On GD 17, 19, and 21, intraperitoneal injection of sterile PBS (0.2 mL per rat) was administered. ABX-ICP group: PGF rats (treated with the same antibiotic mixture as the ABX-PBS group) were subcutaneously injected with EE2 (0.6 mg/kg/day) from GD 17 to GD 21 to induce ICP. On GD 17, 19, and 21, FMT was performed using fecal microbiota isolated from EE2-induced ICP rats. ABX-ICP-RES group: PGF rats (antibiotic-treated) were subcutaneously injected with EE2 (0.6 mg/kg/day) from GD 17 to GD 21. FMT was conducted on GD 17, 19, and 21 using fecal microbiota from RES (60 mg/kg)-treated ICP rats.

Fresh fecal samples (100 mg) were collected from donor rats (ICP rats or RES-treated ICP rats) under sterile conditions. Each sample was homogenized in 1 mL of sterile PBS, followed by centrifugation at 800 × g for 5 min at 4 °C. The supernatant was filtered through a 0.22 μm sterile filter to remove large particles and obtain the fecal microbiota suspension. Each recipient PGF rat received 0.2 mL of the prepared suspension via oral gavage on the scheduled FMT days. All rats in the FMT experiment were euthanized on GD 21, and serum, liver, intestinal tissues, and fecal samples were collected using the same methods as described in Section 2.3.

### 2.5 Serum biochemical and hepatic inflammatory mediator analysis

Serum levels of LPS, IL-1β, and TBil were measured using commercial ELISA kits according to the manufacturers’ instructions. Serum activities of aspartate aminotransferase (AST), alanine aminotransferase (ALT), alkaline phosphatase (ALP), and glutathione S-transferase (GST) were detected using an automatic biochemical analyzer (Hitachi 7600, Tokyo, Japan). For hepatic MCP-1 detection, frozen liver tissue was homogenized in ice-cold PBS (10%, w/v) and centrifuged at 12,000 × g for 20 min at 4 °C. The supernatant was collected, and MCP-1 concentration was determined using an ELISA kit following the manufacturer’s protocol. All experiments were performed in triplicate to ensure reproducibility.

### 2.6 Histopathological examination

Liver and intestinal tissues fixed in 4% paraformaldehyde were dehydrated through a graded ethanol series, cleared in xylene, and embedded in paraffin. Paraffin-embedded tissues were sectioned into 5 μm-thick slices, which were then stained with hematoxylin and eosin (H&E). Stained sections were observed under a light microscope (Olympus BX53, Tokyo, Japan). Histopathological changes were evaluated by two independent pathologists in a blinded manner. For liver tissues, the evaluation focused on inflammatory cell infiltration, hepatocellular necrosis, and structural disruption. For intestinal tissues, villus height, crypt depth, and mucosal integrity were assessed to determine intestinal barrier function.

### 2.7 Hepatic bile acid profiling by ultra-high performance liquid chromatography-tandem mass spectrometry

Frozen liver tissue (50 mg) was homogenized in 1 mL of methanol and sonicated for 30 min on ice. After centrifugation at 15,000 × g for 15 min at 4 °C, the supernatant was collected, evaporated to dryness under a stream of nitrogen gas, and reconstituted in 100 μL of methanol. The resulting solution was filtered through a 0.22 μm sterile membrane filter prior to UHPLC-MS/MS analysis.

Bile acid separation was carried out on a UHPLC system (Thermo Scientific Ultimate 3000, Waltham, MA, USA) equipped with a C18 column (2.1 × 100 mm, 1.7 μm; Waters, Milford, MA, USA). The mobile phase consisted of acetonitrile (Phase A) and 0.1% formic acid in water (Phase B). A gradient elution program was applied as follows: 0–5 min, 20% A; 5–15 min, 20–60% A; 15–20 min, 60–90% A; 20–22 min, 90% A; 22–23 min, 90–20% A; 23–28 min, 20% A. The flow rate was set at 0.3 mL/min, and the column temperature was maintained at 40 °C.

Mass spectrometric detection was performed using a triple quadrupole mass spectrometer (Thermo Scientific TSQ Quantiva, Waltham, MA, USA) with electrospray ionization in negative mode. The instrument parameters were as follows: spray voltage, 3500 V; capillary temperature, 320 °C; sheath gas pressure, 35 arbitrary units; auxiliary gas pressure, 10 arbitrary units. Quantitative analysis of individual bile acids—including glycocholic acid (GCA), chenodeoxycholic acid (CDCA), ursodeoxycholic acid (UDCA), tauroursodeoxycholic acid (TUDCA), taurocholic acid (TCA), cholic acid (CA), and taurodeoxycholic acid (TDCA)—was conducted in selected reaction monitoring mode. Calibration curves were constructed using authentic bile acid standards (Sigma-Aldrich) to determine the concentrations of target bile acids in the liver samples. All analyzes were performed in triplicate.

### 2.8 Gut microbiota analysis by 16S rRNA gene sequencing

Total microbial DNA was extracted from frozen fecal samples (200 mg) using the QIAamp Fast DNA Stool Mini Kit according to the manufacturer’s instructions. DNA concentration and purity were determined using a NanoDrop 2000 spectrophotometer (Thermo Scientific), and DNA integrity was verified by 1% agarose gel electrophoresis. The V3–V4 hypervariable region of the bacterial 16S rRNA gene was amplified using the forward primer 341F (5’-CCTAYGGGRBGCASCAG-3’) and the reverse primer 806R (5’-GGACTACNNGGGTATCTAAT-3’). PCR reactions were performed in a 25 μL volume containing 12.5 μL of 2× Taq PCR MasterMix, 1 μL of each primer (10 μM), 2 μL of template DNA, and 8.5 μL of sterile water. The PCR program was as follows: initial denaturation at 95 °C for 5 min; 30 cycles of denaturation at 95 °C for 30 s, annealing at 55 °C for 30 s, and extension at 72 °C for 45 s; and a final extension at 72 °C for 10 min. PCR products were purified using the Agencourt AMPure XP Kit to remove non-specific amplicons. Purified PCR products were quantified using a Qubit 3.0 fluorometer (Thermo Scientific), and equimolar amounts of amplicons from each sample were pooled. The pooled library was sequenced on the Illumina MiSeq platform (Illumina, San Diego, CA, USA) using the MiSeq Reagent Kit v3 (600 cycles) according to the manufacturer’s protocols. Raw sequencing reads were processed using QIIME 2 software (version 2022.8). Low-quality reads (quality score < 20, read length < 200 bp) were filtered out, and chimeric sequences were removed using the DADA2 plugin. The remaining high-quality sequences were clustered into operational taxonomic units at 97% sequence similarity. Taxonomic classification of OTUs was performed against the Silva database (version 138) using the q2-feature-classifier plugin. Alpha diversity indices, including ACE, Chao1 (for microbial richness), Simpson, and Shannon (for microbial evenness), were calculated using the q2-diversity plugin. Beta diversity was analyzed using principal coordinate analysis based on Bray–Curtis dissimilarity matrices to visualize differences in microbial community structure among groups. Relative abundances of gut microbiota at the phylum and genus levels were calculated and compared between groups.

### 2.9 Western blotting analysis

Total protein was extracted from frozen liver tissue (100 mg) by homogenization in RIPA lysis buffer (Beyotime, Shanghai, China) containing protease and phosphatase inhibitors (Roche, Basel, Switzerland) on ice for 30 min. The homogenate was centrifuged at 12,000 × g for 20 min at 4 °C, and the resulting supernatant was collected as the total protein extract. Protein concentration was determined using a BCA Protein Assay Kit (Thermo Scientific, USA). Equal amounts of protein (50 μg per lane) were separated by 10% sodium dodecyl sulfate–polyacrylamide gel electrophoresis and subsequently transferred onto polyvinylidene fluoride membranes (Millipore, Billerica, MA, USA). The membranes were blocked with 5% non-fat milk in Tris-buffered saline containing 0.1% Tween 20 (TBST) for 1 h at room temperature, followed by incubation with specific primary antibodies at 4 °C overnight. The primary antibodies were used at the following dilutions: FXR (1:1000), SHP (1:1000), BSEP (1:800), UGT2B4 (1:500), CYP1A1 (1:1200), NTCP (1:1000), and GAPDH (1:5000). After primary antibody incubation, the membranes were washed three times with TBST (10 min per wash) and then incubated with horseradish peroxidase–conjugated secondary antibodies (anti-rabbit or anti-mouse IgG, both at 1:2000 dilution) for 1 h at room temperature. Protein bands were visualized using an enhanced chemiluminescence detection system (Thermo Scientific, USA), and band intensities were quantified using ImageJ software (National Institutes of Health, Bethesda, MD, USA). GAPDH served as the internal loading control for normalizing target protein expression levels. All Western blotting analyzes were performed in three independent experiments.

### 2.10 Statistical analysis

All experimental data were expressed as the mean ± standard deviation. Statistical analysis was performed using SPSS 22.0 software (IBM, Armonk, NY, USA) and GraphPad Prism 8.0 software (GraphPad Software, San Diego, CA, USA). Differences between multiple groups were analyzed using one-way analysis of variance followed by Tukey’s *post-hoc* test. A *P*-value < 0.05 was considered statistically significant.

## 3 Results

### 3.1 RES alleviates systemic inflammation and intestinal barrier dysfunction in ICP rats

**Figure 1:**
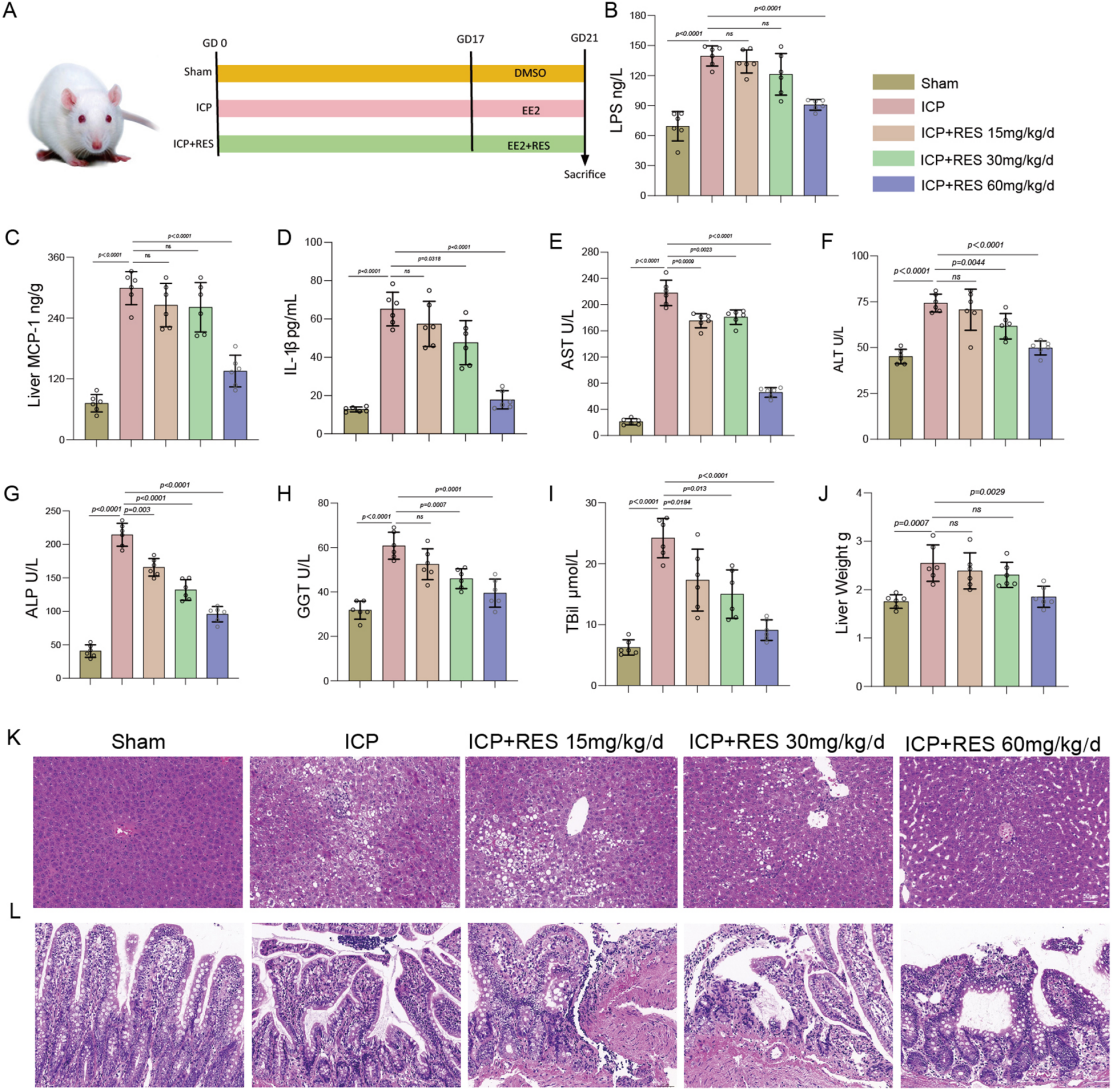
RES alleviated systemic inflammation and intestinal barrier dysfunction in ICP rats. **(A)** Experimental timeline showing EE2-induced ICP model establishment and RES treatment protocol. **(B)** Serum LPS levels. **(C)** Hepatic MCP-1 levels. **(D)** Hepatic IL-1β concentrations. **(E)** Serum AST activity. **(F)** Serum ALT activity. **(G)** Serum ALP activity. **(H)** GST levels. **(I)** Serum TBil concentrations. **(J)** Liver weight. **(K)** Representative H&E-stained liver sections. Scale bar: 50 μm. **(L)** Representative H&E-stained intestinal sections. Scale bar: 50 μm. Data represent mean ± SEM (n=6/group).

To evaluate the therapeutic potential of RES against ICP, we established a rat model through subcutaneous administration of EE2 starting from GD 17. Animals received concurrent RES treatment at doses of 15, 30, or 60 mg/kg/day. Sham-operated rats and ICP rats treated with vehicle served as controls (**Figure 1A**). Initial assessment of systemic inflammation and intestinal barrier integrity through serum LPS measurement revealed significantly elevated LPS levels in the ICP group compared to Sham controls (**Figure 1B**). RES administration produced a dose-dependent reduction in LPS concentrations, with the most pronounced effect at 60 mg/kg/day, indicating attenuation of systemic inflammation. Evaluation of hepatic pro-inflammatory mediators showed marked upregulation of MCP-1 in the ICP group, which was dose-dependently suppressed by RES treatment (**Figure 1C**). Similarly, IL-1β concentrations were elevated in ICP rats and significantly decreased by RES in a dose-dependent manner (**Figure 1D**), confirming the anti-inflammatory efficacy of RES in the liver. Liver function assessment demonstrated substantially elevated activities of AST, ALT, and ALP in the ICP group compared to Sham controls (**Figures 1E–G**), indicating severe hepatic injury. RES treatment dose-dependently reduced these enzyme activities, with the 60 mg/kg/day group exhibiting values closest to Sham levels. GST, a marker of hepatic detoxification capacity, was decreased in the ICP group and restored dose-dependently by RES (**Figure 1H**), collectively indicating improved liver function. TBil, a key cholestasis marker, was significantly elevated in the ICP group and reduced in a dose-dependent manner following RES treatment (**Figure 1I**), suggesting alleviation of cholestasis. Liver weight, which reflects pathological changes, was increased in the ICP group and normalized by RES administration (**Figure 1J**), consistent with histological improvement. Histopathological evaluation via H&E staining revealed normal hepatic architecture in the Sham group, while the ICP group exhibited severe injury characterized by inflammatory infiltration and structural disruption. RES treatment dose-dependently improved these alterations, with near-normal morphology observed at the highest dose (**Figure 1K**). Examination of intestinal histology showed intact villi and mucosal structure in the Sham group, contrasted with villus atrophy and mucosal damage in the ICP group. RES administration dose-dependently restored intestinal integrity (**Figure 1L**), demonstrating protection of the intestinal barrier. These results indicate that RES treatment dose-dependently attenuates systemic and hepatic inflammation, improves liver function, alleviates cholestasis, and mitigates histopathological injury in both liver and intestine of ICP rats, supporting its potential as a therapeutic agent for ICP.

### 3.2 RES restores disrupted hepatic bile acid metabolism in ICP rats

**Figure 2:**
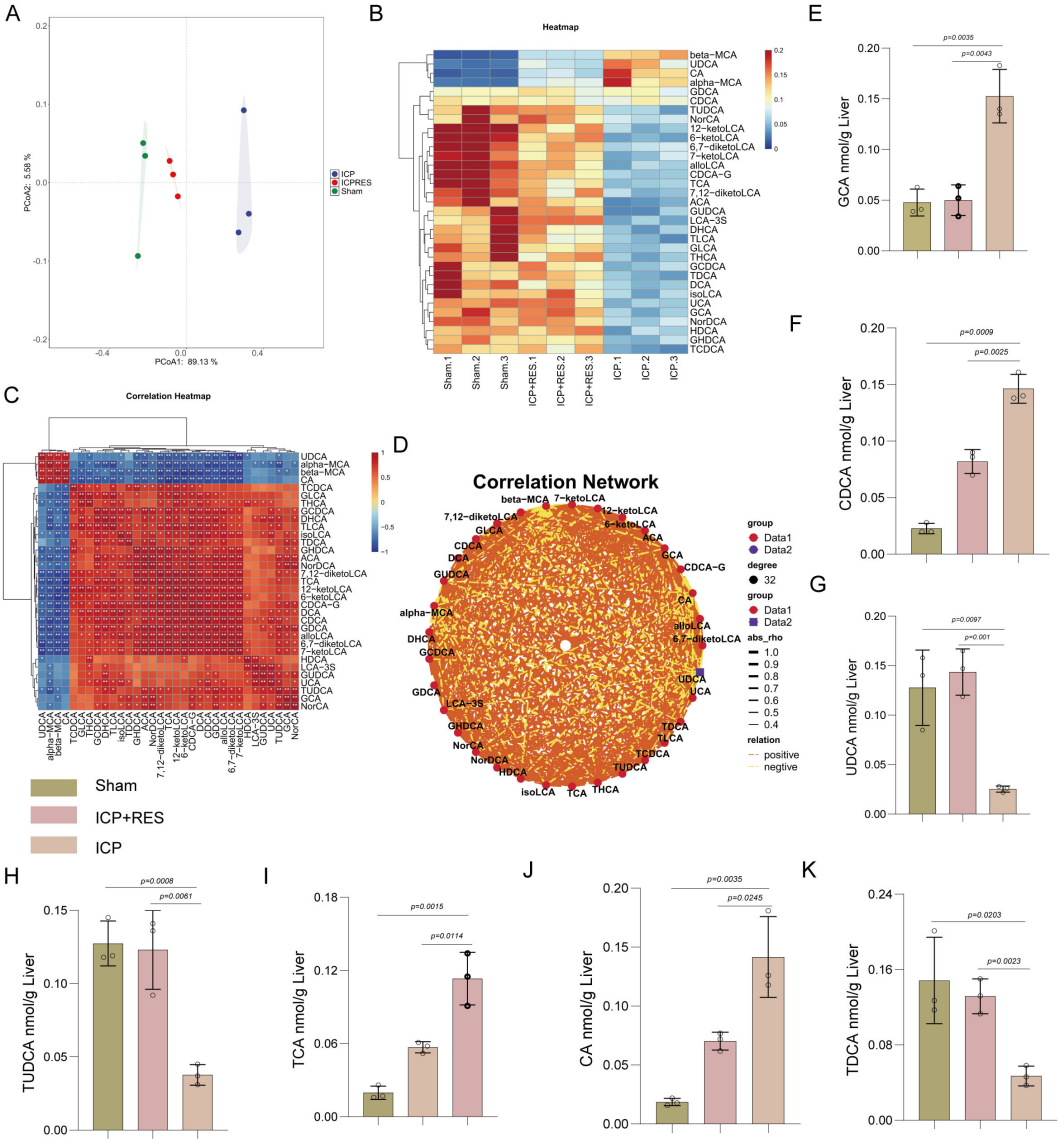
RES treatment attenuates ICP-induced alterations in hepatic bile acid metabolism. **(A)** Principal component analysis (PCA) score plot showing distinct clustering of sham, ICP, and ICP+RES groups. **(B)** Hierarchical clustering heatmap displaying abundance patterns of individual bile acids across experimental groups. **(C)** Correlation heatmap illustrating interactions among different bile acid species. **(D)** Correlation network visualization with orange edges representing positive correlations. **(E-K)** Quantitative analysis of specific bile acids: **(E)** glycocholic acid (GCA), **(F)** chenodeoxyglycocholic acid (CDCA), **(G)** ursodeoxycholic acid (UDCA), **(H)** tauroursodeoxycholic acid (TUDCA), **(I)**,taurocholic acid (TCA), **(J)** cholic acid (CA), and **(K)** taurodeoxycholic acid (TDCA). Data are presented as mean ± SEM (n=3/group).

Comprehensive analysis of hepatic bile acid metabolism using principal component analysis revealed clear separation among Sham, ICP, and RES-treated ICP groups (**Figure 2A**), indicating distinct metabolic profiles. Hierarchical clustering analysis demonstrated differential abundance patterns of individual bile acids, with metabolites clustering into modules showing pronounced variations (**Figure 2B**). Correlation heatmap analysis revealed complex regulatory networks with robust positive and negative interactions among bile acids (**Figure 2C**), visualized through a correlation network showing dense positive connections (orange edges) indicating coordinated regulation of bile acid metabolism (**Figure 2D**). Quantification of specific bile acids showed significantly elevated levels of GCA in the ICP group compared to Sham controls, which was dose-dependently reduced by RES treatment (**Figure 2E**). Similarly, CDCA was increased in the ICP group and suppressed by RES (**Figure 2F**), indicating that RES limits primary bile acid accumulation. Among secondary bile acids, UDCA was decreased in the ICP group and significantly elevated by RES treatment (**Figure 2G**). Its conjugated form, TUDCA, showed parallel reduction in ICP rats and restoration by RES (**Figure 2H**). The primary conjugated bile acid TCA was elevated in the ICP group and decreased following RES administration (**Figure 2I**), while CA was increased in ICP rats and reduced by RES (**Figure 2J**). TDCA, a secondary conjugated bile acid, was also decreased in the ICP group and normalized by RES (**Figure 2K**). These findings demonstrate that RES treatment effectively reverses the ICP-induced imbalance between elevated primary and reduced secondary bile acids in the liver.

### 3.3 RES restores gut microbiota diversity and composition in ICP rats

**Figure 3:**
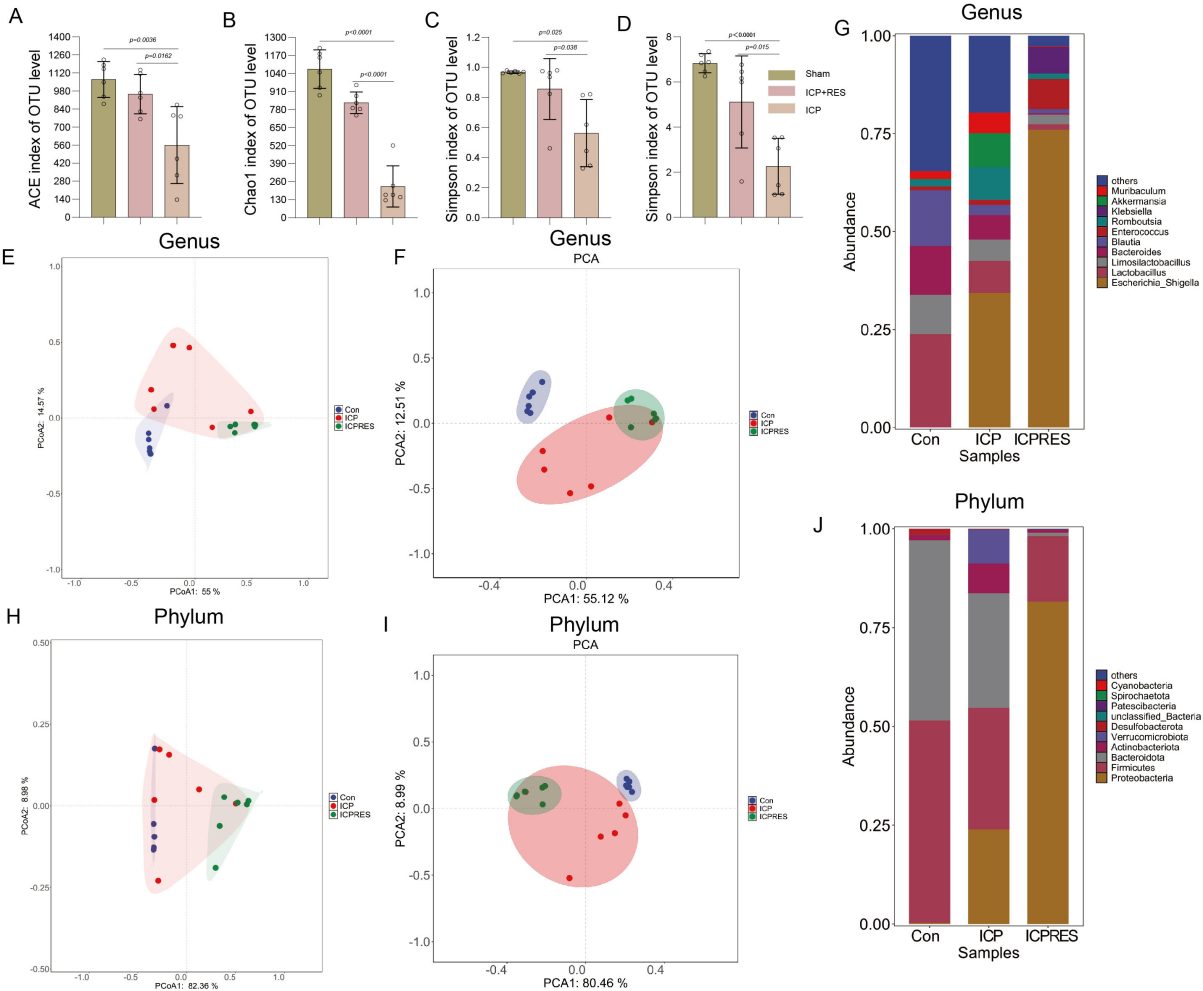
Resveratrol restores gut microbiota diversity and composition in ICP rats. **(A, B)** Alpha diversity assessed by ACE index **(A)** and Chao index **(B)**, demonstrating species richness. **(C, D)** Alpha diversity assessed by Simpson index **(C)** and corresponding statistical analysis **(D)**, reflecting species evenness. **(E, F)** Principal coordinate analysis (PCoA) at the genus level based on unweighted **(E)** and weighted **(F)** UniFrac distances, showing structural separation of microbial communities. **(G)** Relative abundance of major bacterial genera across different groups. **(H, I)** PCoA at the phylum level using unweighted **(H)** and weighted **(I)** UniFrac distances. **(J)** Phylum-level compositional changes in gut microbiota among experimental groups. Data in bar graphs are presented as mean ± SEM (n=6 per group) (one-way ANOVA with Tukey’s *post-hoc* test).

Analysis of gut microbiota diversity revealed significantly reduced ACE and Chao1 indices in the ICP group compared to Sham controls, indicating impaired species richness. RES treatment increased both indices (**Figures 3A, B**), demonstrating partial restoration of microbial richness. The Simpson index, measuring species evenness, was also reduced in the ICP group and elevated by RES treatment (**Figures 3C, D**), suggesting improved community evenness. Principal coordinate analysis at the genus level revealed clear separation among Sham, ICP, and ICP+RES groups (**Figures 3E, F**), indicating structural shifts in microbial composition. Stacked bar plot analysis at the genus level (**Figure 3G**) illustrated abundance alterations in major genera including Lactobacillus, Bacteroides, and Allobaculum in the ICP group, which were reversed by RES treatment. PCoA at the phylum level (**Figures 3H, I**) confirmed distinct clustering among groups. The phylum-level composition analysis (**Figure 3J**) revealed ICP-induced alterations in abundances of Firmicutes, Bacteroidota, and Proteobacteria, largely normalized by RES treatment. These results indicate that ICP disrupts gut microbiota diversity and community structure, and that RES treatment effectively restores these parameters, suggesting microbiota modulation contributes to the therapeutic effects of RES.

### 3.4 Gut microbiota mediates the therapeutic effects of RES in ICP

**Figure 4:**
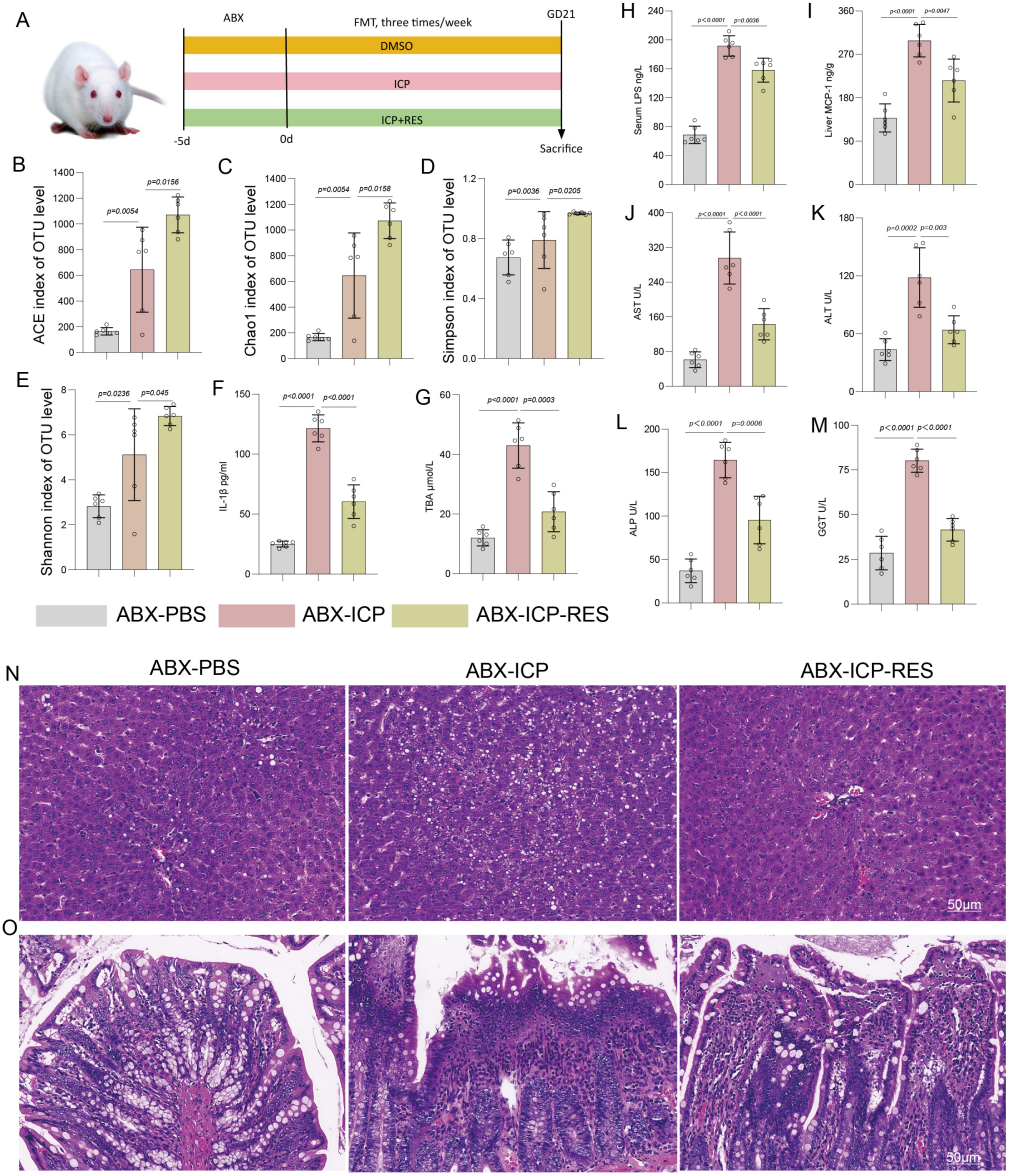
Fecal microbiota transplantation from RES-treated donors alleviated disease phenotype in pseudogerm-free ICP rats. **(A)** Schematic diagram of the experimental design for antibiotic-induced pseudogerm-free model establishment and fecal microbiota transplantation (FMT). **(B-E)** Gut microbiota alpha diversity indices: **(B)** ACE index, **(C)** Chao1 index, **(D)** Simpson index, and **(E)** Shannon index. **(F)** Serum interleukin-1β (IL-1β) levels. **(G)** Serum total bilirubin (TBil) concentrations. **(H)** Serum lipopolysaccharide (LPS) levels. **(I)** Hepatic monocyte chemoattractant protein-1 (MCP-1) expression. **(J-M)** Liver function parameters: **(J)** aspartate aminotransferase (AST), **(K)** alanine aminotransferase (ALT), **(L)** alkaline phosphatase (ALP), and **(M)** glutathione S-transferase (GST) activities. **(N)** Representative H&E-stained liver sections. **(O)** Representative H&E-stained intestinal sections. Data are presented as mean ± SEM (n=6 per group).

To investigate gut microbiota involvement in RES-mediated protection against ICP, we established a PGF rat model via ABX treatment. FMT was performed three times weekly using donor microbiota from ICP rats (ABX-ICP group) or RES-treated ICP rats (ABX-ICP-RES group). ABX rats receiving PBS served as controls (ABX-PBS group). All animals were sacrificed at GD 21 (**Figure 4A**). Alpha diversity analysis showed significantly higher ACE (**Figure 4B**) and Chao1 (**Figure 4C**) indices in both ABX-ICP and ABX-ICP-RES groups compared to the ABX-PBS group, confirming successful microbial colonization. Similarly, Simpson (**Figure 4D**) and Shannon (**Figure 4E**) indices were elevated in FMT groups, demonstrating restored microbial diversity. Assessment of systemic inflammation and cholestasis revealed significantly increased IL-1β levels in the ABX-ICP group, which were markedly reduced in the ABX-ICP-RES group (**Figure 4F**). TBil (TBA) concentrations were elevated in ABX-ICP rats and significantly attenuated by RES-modulated FMT (**Figure 4G**), indicating alleviation of systemic inflammation and cholestasis. Liver function evaluation showed substantially higher serum LPS levels in the ABX-ICP group compared to ABX-PBS controls, which were normalized in the ABX-ICP-RES group (**Figure 4H**). Hepatic MCP-1 expression was elevated in the ABX-ICP group and reduced following RES-modulated FMT (**Figure 4I**). Activities of AST (**Figure 4J**), ALT (**Figure 4K**), ALP (**Figure 4L**), and GST (**Figure 4M**) were all increased in the ABX-ICP group and significantly improved in the ABX-ICP-RES group, demonstrating restored hepatic function. Histopathological assessment revealed normal liver architecture in the ABX-PBS group, severe inflammatory infiltration and structural disruption in the ABX-ICP group, and marked improvement in the ABX-ICP-RES group (**Figure 4N**). Intestinal histology showed preserved villous structure in ABX-PBS rats, villous atrophy and mucosal damage in ABX-ICP rats, and substantial restoration of intestinal integrity in the ABX-ICP-RES group (**Figure 4O**). These findings demonstrate that transplantation of RES-modulated microbiota recapitulates the therapeutic benefits of RES in PGF ICP rats, indicating gut microbiota plays a critical role in RES efficacy against ICP.

### 3.5 FMT from RES-treated donors restores hepatic bile acid metabolism

**Figure 5:**
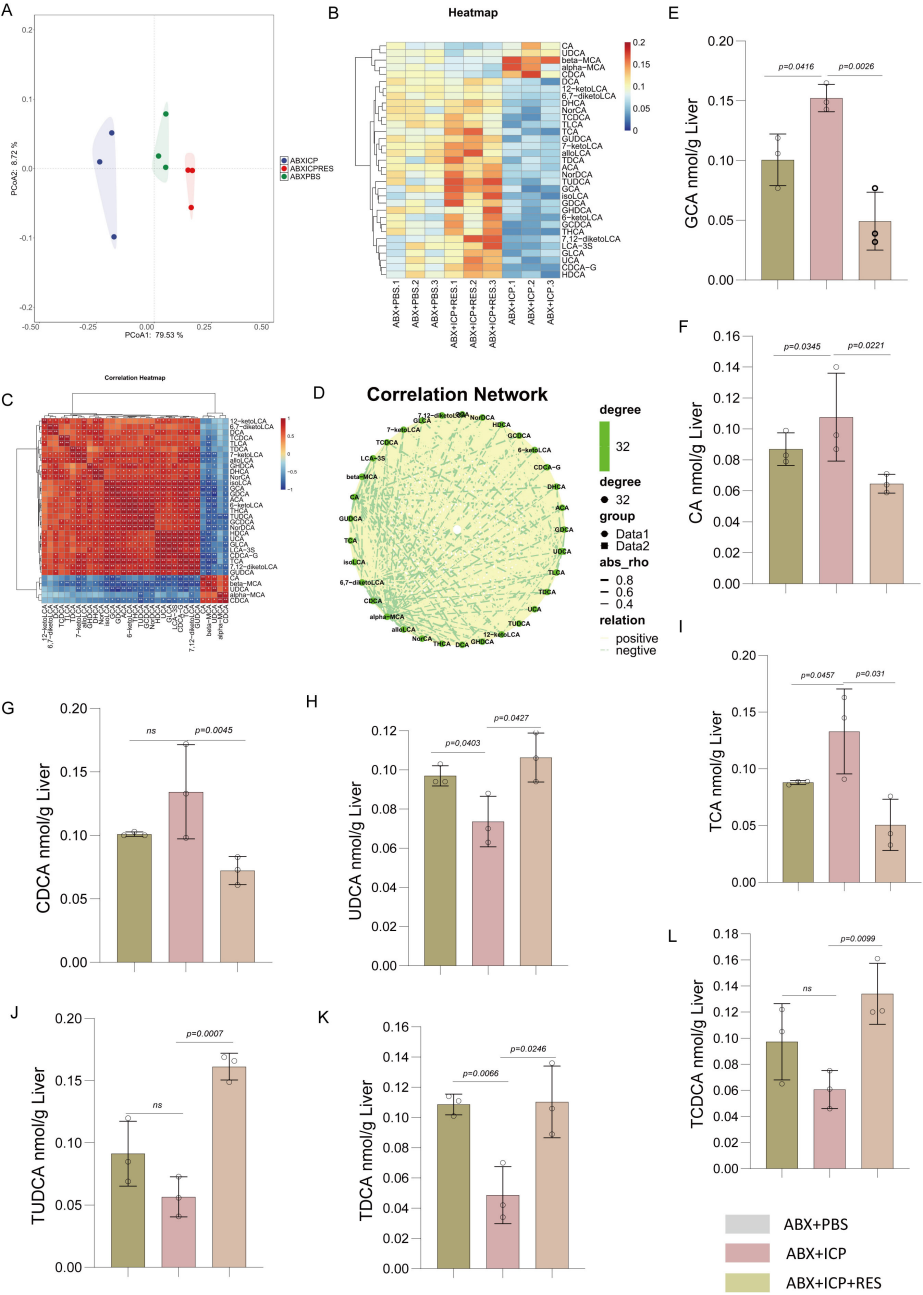
Fecal microbiota transplantation from RES-treated donors restores hepatic bile acid metabolism in pseudogerm-free ICP rats. **(A)** Principal component analysis (PCA) score plot showing distinct clustering of ABX+PBS, ABX+ICP, and ABX+ICP+RES groups. **(B)** Hierarchical clustering heatmap displaying abundance patterns of individual bile acids across experimental groups. **(C)** Correlation heatmap illustrating interactions among different bile acid species. **(D)** Correlation network visualization with green edges representing positive correlations. **(E-G)** Primary bile acid quantification: **(E)** glycocholic acid (GCA), **(F)** cholic acid (CA), and **(G)** chenodeoxycholic acid (CDCA). **(H)** Secondary bile acid ursodeoxycholic acid (UDCA) levels. **(I)** taurocholic acid (TCA), **(J)** tauroursodeoxycholic acid (TUDCA), **(K)** taurodeoxycholic acid (TDCA), and **(L)** taurochenodeoxycholic acid (TCDCA).Data are presented as mean ± SEM (n=3 per group).

Analysis of hepatic bile acid metabolism in ABX+PBS, ABX+ICP, and ABX+ICP+RES groups using PCA revealed clear separation among groups (**Figure 5A**), indicating distinct metabolic profiles following FMT. Hierarchical clustering heatmap illustrated differential abundance patterns of individual bile acids (**Figure 5B**). Correlation analysis revealed complex interactions within the bile acid network (**Figure 5C**), visualized through a correlation network showing dense positive connections (green edges) suggesting coordinated regulation (**Figure 5D**). Quantification showed significantly elevated levels of GCA (**Figure 5E**), CA (**Figure 5F**), CDCA (**Figure 5G**), and TCA (**Figure 5I**) in the ABX+ICP group, which were markedly reduced by RES-modulated FMT. Secondary bile acids including UDCA (**Figure 5H**), TUDCA (**Figure 5J**), TDCA (**Figure 5K**), and TCDCA (**Figure 5L**) were decreased in the ABX+ICP group and restored toward normal levels following RES-modulated FMT. These results demonstrate that RES-modulated microbiota reverses the ICP-induced imbalance between primary and secondary bile acids, further supporting gut–liver axis involvement in the therapeutic mechanism of RES.

### 3.6 RES restores hepatic bile acid homeostasis via the FXR signaling pathway

**Figure 6:**
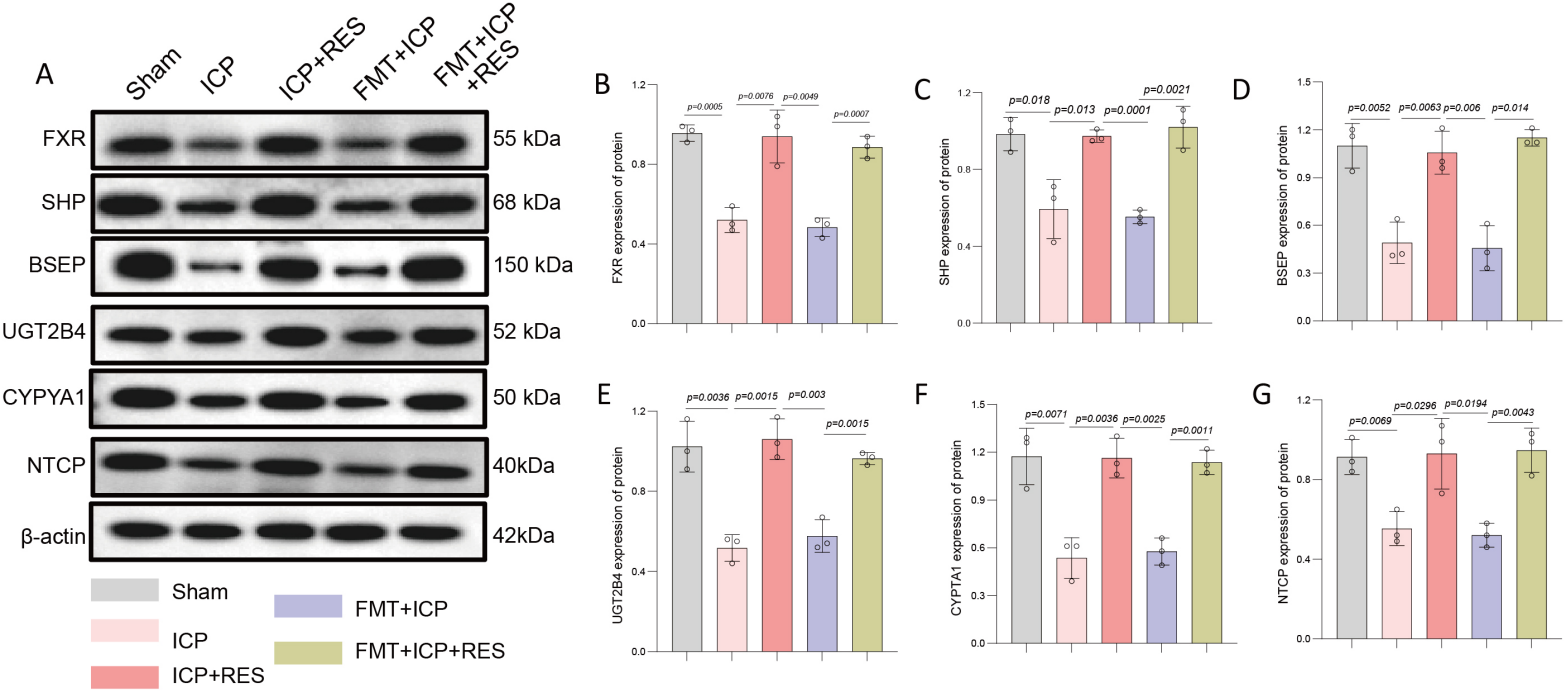
RES regulates key molecules in the hepatic bile acid metabolism pathway through FXR-mediated signaling. **(A)** Representative Western blot images of proteins involved in bile acid metabolism and transport. **(B-G)** Quantitative analysis of protein expression levels: **(B)** Farnesoid X receptor (FXR), **(C)** Small heterodimer partner (SHP), **(D)** Bile salt export pump (BSEP), **(E)** Uridine 5’-diphospho-glucuronosyltransferase 2B4 (UGT2B4), **(F)** Cytochrome P450 family 1 subfamily A member 1 (CYP1A1), and **(G)** Sodium taurocholate cotransporting polypeptide (NTCP). Data are presented as mean ± SEM (n=3 per group).

Investigation of the molecular mechanisms underlying RES-mediated improvement in bile acid metabolism analyzed protein expression of key regulators by Western blotting (**Figure 6A**). Targets included FXR, SHP, BSEP, UGT2B4, CYP1A1, and NTCP. FXR expression was significantly downregulated in ICP and FMT+ICP groups compared to the Sham group, but markedly increased in both ICP+RES and FMT+ICP+RES groups (**Figure 6B**). SHP, a downstream FXR target, showed a similar pattern with decreased expression in ICP and FMT+ICP groups and elevation in RES-treated groups (**Figure 6C**). BSEP expression was downregulated in ICP and FMT+ICP groups and robustly upregulated following RES treatment (**Figure 6D**). UGT2B4 and CYP1A1 were reduced in ICP and FMT+ICP groups and significantly increased in RES-treated groups (**Figures 6E, F**). NTCP expression was downregulated in ICP and FMT+ICP groups and restored by RES treatment (**Figure 6G**). These results indicate that RES treatment, both direct and via microbiota modulation, restores expression of key bile acid metabolism proteins through FXR-mediated signaling pathway activation, improving bile acid homeostasis in ICP rats.

## 4 Discussion

This study provides compelling evidence that resveratrol confers significant protection against estrogen-induced intrahepatic cholestasis of pregnancy through a coordinated multi-mechanistic approach involving modulation of the gut-liver axis, suppression of inflammatory responses, and restoration of bile acid homeostasis primarily via FXR signaling pathway activation. Our findings not only confirm the hepatoprotective properties of RES but also elucidate previously unrecognized mechanisms underlying its therapeutic efficacy in ICP, establishing its potential as a viable candidate for clinical translation.

The anti-inflammatory properties of RES observed in our ICP model represent a crucial component of its therapeutic mechanism. RES administration significantly reduced serum LPS levels, indicating enhancement of intestinal barrier function and attenuation of bacterial translocation. This finding is particularly relevant in cholestatic liver diseases, where gut-derived endotoxemia contributes substantially to disease progression (11, 12). The elevated LPS levels in ICP rats likely reflect intestinal barrier compromise, facilitating the entry of bacterial products into the portal circulation and subsequent initiation of hepatic inflammation. However, this study did not assess fetal outcomes (litter size, weight, length, or malformations) — a limitation. The RES doses used (15, 30, 60 mg/kg/day) translate to ~145, 290, 580 mg/day for a 60 kg adult. RES crosses the placenta and has weak estrogenic activity; its safety in human pregnancy is unknown. Further reproductive toxicity studies are needed.

The concomitant reduction in hepatic MCP-1 and IL-1β further substantiates the anti-inflammatory efficacy of RES in the ICP context. These cytokines function as critical mediators in the inflammatory cascades of cholestatic liver injury, with MCP-1 promoting monocyte infiltration and IL-1β amplifying inflammatory responses through NLRP3 inflammasome activation (13). The dose-dependent suppression of these pro-inflammatory mediators by RES treatment indicates a potent anti-inflammatory effect contributing to its overall hepatoprotection.

In this study, 16S rRNA sequencing data reveal that ICP induces substantial gut microbiota dysbiosis characterized by diminished microbial diversity and altered community structure. RES treatment effectively normalized these perturbations, particularly influencing genera including *Lactobacillus* and *Bacteroides*, which play established roles in bile acid metabolism and intestinal barrier maintenance. The restoration of microbial richness and evenness indices by RES treatment indicates a broad-spectrum modulatory effect on the gut ecosystem. The specific increase in *Lactobacillus* abundance following RES treatment is noteworthy, as this genus has been associated with enhanced gut barrier function and reduced bacterial translocation in various liver diseases (14, 15). Similarly, the normalization of Bacteroides populations by RES may contribute to bile acid metabolism, as members of this genus express bile salt hydrolases that catalyze the deconjugation of primary bile acids (16). The intestinal histopathological improvements observed in RES-treated rats, including restoration of villus architecture and mucosal integrity, provide morphological correlates to the functional enhancements in gut barrier function. These findings align with previous reports demonstrating that RES ameliorates intestinal inflammation and barrier dysfunction in models of inflammatory bowel disease (17).

Further, FMT experiments provided compelling evidence that the gut microbiota mediates RES-induced protection. Transferring RES-modulated microbiota conferred protection comparable to direct RES administration, significantly improving systemic inflammation, cholestasis, liver function, and histopathology in the ABX-ICP-RES group despite identical injury. This demonstrates that RES remodels a microbiota with intrinsic protective properties against ICP pathogenesis. These findings indicate that microbiota modulation is a primary mechanism of RES, suggesting its clinical potential lies in inducing durable microbial changes for sustained therapeutic benefits.

Comprehensive bile acid profiling reveals that RES treatment effectively rectifies the ICP-induced imbalance between primary and secondary bile acids. The accumulation of primary bile acids (GCA, CA, CDCA, TCA) in ICP rats and subsequent normalization by RES treatment underscores the efficacy of RES in restoring bile acid homeostasis. Particularly notable is the increased abundance of beneficial secondary bile acids, including UDCA and its conjugated derivatives, which are recognized for their hepatoprotective properties. The elevated levels of UDCA and TUDCA following RES treatment are clinically relevant, as UDCA constitutes the current first-line therapy for ICP and other cholestatic liver diseases (18). These secondary bile acids have been demonstrated to ameliorate endoplasmic reticulum stress and mitochondrial dysfunction in hepatocytes, providing plausible mechanisms for their therapeutic benefits (19, 20). The FMT experiments further confirmed that bile acid metabolic improvements could be transferred through microbiota transplantation, reinforcing the role of gut microbes in this process. Gut microbiota contribute to bile acid metabolism through various enzymatic activities, including deconjugation, dehydroxylation, and epimerization reactions (21). The restoration of secondary bile acid levels in the ABX-ICP-RES group indicates that RES-modulated microbiota enhances these biotransformation pathways.

At the molecular level, our study identifies FXR signaling as a principal pathway mediating the beneficial effects of RES on bile acid homeostasis. The significant upregulation of FXR and its downstream targets in RES-treated groups provides mechanistic insight into RES-mediated improvement of bile acid metabolism. FXR activation typically reduces bile acid synthesis through SHP-mediated CYP7A1 suppression and enhances bile acid export via BSEP induction (22). The simultaneous upregulation of UGT2B4 and CYP1A1 suggests that RES additionally enhances bile acid detoxification and alternative elimination pathways, representing complementary protective mechanisms. UGT2B4 facilitates bile acid glucuronidation, increasing their water solubility and renal excretion (23), while CYP1A1 contributes to hydroxylation reactions that enhance bile acid elimination (24). The regulation of NTCP expression by RES warrants specific consideration. While NTCP downregulation in cholestasis generally represents an adaptive response to reduce hepatic bile acid uptake, its restoration by RES treatment implies more complex regulatory mechanisms. Contemporary investigations reveal that persistent NTCP suppression promise hepatic regeneration and function, indicating that controlled NTCP expression is essential for maintaining bile acid homeostasis (25).

Although our findings establish that RES modulates FXR signaling in conjunction with gut microbiota remodeling, the precise mechanism by which RES influences FXR activity remains to be fully elucidated. RES may act directly as an FXR ligand, as previously suggested for certain polyphenolic compounds, or it may exert its effects indirectly by reshaping the gut microbiota composition, thereby altering the production of endogenous FXR ligands such as secondary bile acids. In the present study, FMT from RES-treated donors recapitulated the activation of FXR signaling in recipient rats, supporting the concept that microbiota-derived factors contribute substantially to the effects of RES. Nevertheless, whether RES directly engages FXR at the molecular level, and the relative contributions of direct versus microbiota-mediated pathways, warrant further investigation using approaches such as FXR reporter assays, co-culture systems, and gnotobiotic models.

Consistent with the findings of Li et al. (26), the present study confirms that resveratrol reverses ICP−associated gut microbial dysbiosis. However, the current work extends those initial observations in several critical aspects. First, fecal microbiota transplantation (FMT) experiments establish a causal relationship between gut microbiota remodeling and the hepatoprotective effects of resveratrol, moving beyond descriptive correlation. Second, the underlying mechanism is identified as FXR−mediated regulation of bile acid transporters (BSEP, NTCP) and detoxifying enzymes (UGT2B4, CYP1A1), which was not previously explored. Third, resveratrol concurrently preserves intestinal barrier integrity and suppresses systemic inflammation, integrating the gut−liver axis into its multi−target action. Collectively, these results build upon the preliminary evidence of Li et al. to provide a causal and mechanistic framework for resveratrol in ICP.

Several study limitations merit acknowledgment. First, while we demonstrate FXR signaling involvement, additional nuclear receptors may contribute to the effects of RES. Second, the specific bacterial taxa responsible for mediating the benefits of RES require precise identification through advanced microbiological techniques. Finally, the potential clinical relevance of our findings necessitates validation through human ICP studies.

In conclusion, our investigation establishes that RES protects against ICP through an integrated network of mechanisms involving gut microbiota modulation, inflammation suppression, and FXR-mediated restoration of bile acid homeostasis. The demonstration that RES-modulated microbiota can transfer therapeutic benefits through FMT provides compelling evidence for the causal role of gut microbes in its mechanism of action. These findings advance our understanding of ICP pathobiology while highlighting the therapeutic potential of targeting the gut-liver axis in cholestatic liver diseases. RES represents a promising multi-mechanistic therapeutic candidate warranting further clinical exploration for ICP management.

## Data Availability

The data generated in this study are publicly available. Data accession numbers CRA039245 and CRA039246 are now publicly released and searchable in GSA database.

## References

[1] Huang X Liao E Chen A Shao Y . Mechanism of mitochondrial dysfunction on placental trophoblastic cells in intrahepatic cholestasis of pregnancy. J Mol Histol. (2025) 56:140. doi: 10.1007/s10735-025-10427-1. PMID: 40278950

[2] Alkurdi A Herrmann J Bikmukhametov D Tschope R . Biliary cast syndrome and secondary sclerosing cholangitis in critically ill patient after long-term treatment in the intensive care unit. Case Rep Gastroenterol. (2024) 18:260–5. doi: 10.1159/000537957. PMID: 38737441 PMC11087035

[3] Zeng W Hou Y Gu W Chen Z . Proteomic biomarkers of intrahepatic cholestasis of pregnancy. Reprod Sci. (2024) 31:1573–85. doi: 10.1007/s43032-023-01437-z. PMID: 38177949 PMC11111573

[4] Pezzino S Sofia M Faletra G Mazzone C Litrico G La Greca G . Gut-liver axis and non-alcoholic fatty liver disease: A vicious circle of dysfunctions orchestrated by the gut microbiome. Biol (Basel). (2022) 11(11):1622. doi: 10.3390/biology11111622. PMID: 36358323 PMC9687983

[5] Termite F ArChilei S D'Ambrosio F Petrucci L Viceconti N Iaccarino R . Gut microbiota at the crossroad of hepatic oxidative stress and MASLD. Antioxidants (Basel). (2025) 14(1):56. doi: 10.3390/antiox14010056. PMID: 39857390 PMC11759774

[6] Hu H Shao W Liu Q Liu N Wang Q Xu J . Gut microbiota promotes cholesterol gallstone formation by modulating bile acid composition and biliary cholesterol secretion. Nat Commun. (2022) 13:252. doi: 10.1038/s41467-021-27758-8. PMID: 35017486 PMC8752841

[7] Aragones G Gonzalez-Garcia S Aguilar C Richart C Auguet T . Gut microbiota-derived mediators as potential markers in nonalcoholic fatty liver disease. BioMed Res Int. (2019) 2019:8507583. doi: 10.1155/2019/8507583 30719448 PMC6334327

[8] Guo Y Bian X Liu J Zhu M Li L Yao T . Dietary components, microbial metabolites and human health: Reading between the lines. Foods. (2020) 9(8):1045. doi: 10.3390/foods9081045. PMID: 32756378 PMC7466307

[9] Valdes-Sanchez L Moshtaghion SM Caballano-Infantes E Penalver P Rodriguez-Ruiz R Gonzalez-Alfonso JL . Synthesis and evaluation of glucosyl-, acyl- and silyl- resveratrol derivatives as retinoprotective agents: Piceid octanoate notably delays photoreceptor degeneration in a retinitis pigmentosa mouse model. Pharm (Basel). (2024) 17(11):1482. doi: 10.3390/ph17111482 PMC11597447 39598393

[10] Pan Y Xia M Luo J Lu S . Resveratrol promotes wound healing by enhancing angiogenesis via inhibition of ferroptosis. Food Sci Nutr. (2025) 13:e70254. doi: 10.1002/fsn3.70254. PMID: 40330211 PMC12053223

[11] Ibidapo-Obe O Bruns T . Tissue-resident and innate-like T cells in patients with advanced chronic liver disease. JHEP Rep. (2023) 5:100812. doi: 10.1016/j.jhepr.2023.100812. PMID: 37691689 PMC10485156

[12] Li X Xie H Chao JJ Jia YH Zuo J An YP . Profiles and integration of the gut microbiome and fecal metabolites in severe intrahepatic cholestasis of pregnancy. BMC Microbiol. (2023) 23:282. doi: 10.1186/s12866-023-02983-x. PMID: 37784030 PMC10546765

[13] Qiu J Qu Y Li Y Li C Wang J Meng L . Inhibition of RAC1 activator DOCK2 ameliorates cholestatic liver injury via regulating macrophage polarisation and hepatic stellate cell activation. Biol Direct. (2025) 20:21. doi: 10.1186/s13062-025-00612-3. PMID: 39923106 PMC11807328

[14] Yang M Massad K Kimchi ET Staveley-O'Carroll KF Li G . Gut microbiota and metabolite interface-mediated hepatic inflammation. Immunometabolism (Cobham). (2024) 6:e00037. doi: 10.1097/in9.0000000000000037. PMID: 38283696 PMC10810350

[15] Zhang GX Jin L Jin H Zheng GS . Influence of dietary components and traditional Chinese medicine on hypertension: A potential role for gut microbiota. Evid Based Complement Alternat Med. (2021) 2021:5563073. doi: 10.1155/2021/5563073. PMID: 33986817 PMC8079198

[16] Alsultan A Walton G Andrews SC Clarke SR . Staphylococcus aureus FadB is a dehydrogenase that mediates cholate resistance and survival under human colonic conditions. Microbiol (Reading). (2023) 169(3):001314. doi: 10.1099/mic.0.001314. PMID: 36947574 PMC10191381

[17] Martini E Krug SM Siegmund B Neurath MF Becker C . Mend your fences: The epithelial barrier and its relationship with mucosal immunity in inflammatory bowel disease. Cell Mol Gastroenterol Hepatol. (2017) 4:33–46. doi: 10.1016/j.jcmgh.2017.03.007 28560287 PMC5439240

[18] Gonzales E Thompson RJ Jacquemin E . Comment on opinion paper on the diagnosis and treatment of progressive familial intrahepatic cholestasis. JHEP Rep. (2025) 7:101361. doi: 10.1016/j.jhepr.2025.101361. PMID: 40535556 PMC12174988

[19] Zhu J Seo JE Wang S Ashby K Ballard R Yu D . The development of a database for herbal and dietary supplement induced liver toxicity. Int J Mol Sci. (2018) 19(10):2955. doi: 10.3390/ijms19102955. PMID: 30274144 PMC6213387

[20] Xiong W Feng J Liu Y Liu J Fu L Wang Q . ShenQiWan ameliorates renal injury in type 2 diabetic mice by modulating mitochondrial fusion and endoplasmic reticulum stress. Front Pharmacol. (2023) 14:1265551. doi: 10.3389/fphar.2023.1265551. PMID: 38026991 PMC10667480

[21] Wang K Liu Z Tang R Sha Y Wang Z Chen Y . Gallstones in the era of metabolic syndrome: Pathophysiology, risk prediction, and management. Cureus. (2025) 17:e80541. doi: 10.7759/cureus.80541. PMID: 40225487 PMC11993725

[22] Wang XX Xie C Libby AE Ranjit S Levi J Myakala K . The role of FXR and TGR5 in reversing and preventing progression of Western diet-induced hepatic steatosis, inflammation, and fibrosis in mice. J Biol Chem. (2022) 298:102530. doi: 10.1016/j.jbc.2022.102530. PMID: 36209823 PMC9638804

[23] Garcia M Thirouard L Sedes L Monrose M Holota H Caira F . Nuclear receptor metabolism of bile acids and xenobiotics: A coordinated detoxification system with impact on health and diseases. Int J Mol Sci. (2018) 19(11):3630. doi: 10.3390/ijms19113630. PMID: 30453651 PMC6274770

[24] Badawi AF Cavalieri EL Rogan EG . Role of human cytochrome P450 1A1, 1A2, 1B1, and 3A4 in the 2-, 4-, and 16alpha-hydroxylation of 17beta-estradiol. Metabolism. (2001) 50:1001–3. doi: 10.1053/meta.2001.25592 11555828

[25] Ming J Xu Q Gao L Deng Y Yin J Zhou Q . Kinsenoside alleviates 17alpha-ethinylestradiol-induced cholestatic liver injury in rats by inhibiting inflammatory responses and regulating FXR-mediated bile acid homeostasis. Pharm (Basel). (2021) 14(5):452. doi: 10.3390/ph14050452. PMID: 34064649 PMC8151897

[26] Li Z Lei L Ling L Liu Y Xiong Z Shao Y . Resveratrol modulates the gut microbiota of cholestasis in pregnant rats. J Physiol Pharmacol. (2022) 73(2):261–8. doi: 10.26402/jpp.2022.2.09 36193965

